# Executive Control of Sequence Behavior in Pigeons Involves Two Distinct Brain Regions

**DOI:** 10.1523/ENEURO.0296-22.2023

**Published:** 2023-03-03

**Authors:** Lukas Alexander Hahn, Jonas Rose

**Affiliations:** Neural Basis of Learning, Institute of Cognitive Neuroscience, Faculty of Psychology, Ruhr University Bochum, 44801 Bochum, Germany

**Keywords:** behavioral switch, cognition, electrophysiology, executive control, network, pigeon

## Abstract

Executive functions arise from multiple regions of the brain acting in concert. To facilitate such cross-regional computations, the brain is organized into distinct executive networks, like the frontoparietal network. Despite similar cognitive abilities across many domains, little is known about such executive networks in birds. Recent advances in avian fMRI have shown a possible subset of regions, including the nidopallium caudolaterale (NCL) and the lateral part of medial intermediate nidopallium (NIML), that may contribute to complex cognition, forming an action control system of pigeons. We investigated the neuronal activity of NCL and NIML. Single-cell recordings were obtained during the execution of a complex sequential motor task that required executive control to stop executing one behavior and continue with a different one. We compared the neuronal activity of NIML to NCL and found that both regions fully processed the ongoing sequential execution of the task. Differences arose from how behavioral outcome was processed. Our results indicate that NCL takes on a role in evaluating outcome, while NIML is more tightly associated with ongoing sequential steps. Importantly, both regions seem to contribute to overall behavioral output as parts of a possible avian executive network, crucial for behavioral flexibility and decision-making.

## Significance Statement

Executive control is required to reach behavioral goals. This is of particular importance when outcome contingencies change. To stop and switch ongoing behavior requires a neural basis of executive control, like the mammalian frontoparietal network. Using parallel neuronal recordings, we demonstrate that two regions of the pigeon brain participate in the organized execution of two different behavioral sequences and in the switch from one to the other. These results help to elucidate how different aspects of higher cognition are distributed across the brain of an important nonmammalian model species.

## Introduction

The last decades have seen an overhaul of our understanding of the avian brain. The superficial differences between avian and mammalian brains now take a backseat compared with the number of similarities (Jarvis et al., 2005; Güntürkün and Bugnyar, 2016; Stacho et al., 2020; Güntürkün et al., 2021; Nieder, 2017, 2021). The avian telencephalon was once thought to be mostly striatal because it lacks the mammalian separation between gray and white matter along with the organization into cortical layers. Now we know that the ratio between striatum and pallium and even the overall connectivity within ascending sensory systems are comparable between birds and mammals (Jarvis et al., 2005; Stacho et al., 2020). This modern picture of neural similarities rather than differences matches what we know from behavior: the cognition of birds and mammals is surprisingly similar (Emery and Clayton, 2004; Güntürkün and Bugnyar, 2016). At the heart of mammalian cognition lies executive control, a concept that often encompasses working memory, inhibition, and flexibility (Diamond, 2013). Executive control is closely associated with the mammalian prefrontal cortex (PFC; Miller, 2000), but the PFC is not the only cortical structure critical to higher cognition. Frontoparietal areas (e.g., the dorsal anterior cingulate cortex and lateral parietal cortex) form networks that support executive functions (Shenhav et al., 2017).

In the avian brain, however, very little is known about the networks that mediate executive functions. Investigation of the nidopallium caudolaterale (NCL) suggests that the structure is the functional equivalent of mammalian PFC (Güntürkün, 2005; Güntürkün et al., 2021). The NCL is a candidate structure belonging to an executive control network in the avian brain. It is connected to all secondary sensory structures (Kröner and Güntürkün, 1999), receives a rich dopaminergic innervation (Durstewitz et al., 1999; von Eugen et al., 2020), and, importantly, neurophysiological studies report neural correlates in NCL that align with predictions from the PFC. Prominent examples are working memory (Diekamp et al., 2002; Veit et al., 2014; Hahn et al., 2021), reward (Johnston et al., 2017; Packheiser et al., 2021), rules (Veit and Nieder, 2013), decisions (Kalenscher et al., 2005), numerosity (Ditz and Nieder, 2015), categories (Kirsch et al., 2009; Anderson et al., 2020), and executive control (Rose and Colombo, 2005). Recent avian fMRI studies have also revealed that the medial nidopallium/mesopallium (MNM) may be an important associative structure in the avian brain (Behroozi et al., 2020). The MNM receives rich multimodal sensory inputs, is connected to premotor structures, and is reciprocally connected with the NCL (Csillag, 1999; Atoji and Wild, 2012; Shanahan et al., 2013). Lesion/inactivation studies also suggest that this region is involved in sensorimotor learning (Rose, 2000; Horn, 2004) and in sequential behavior (Helduser and Güntürkün, 2012; Helduser et al., 2013; Rook et al., 2021b). The most striking examples of the latter come from songbirds that evolved highly specialized neural circuits for song learning; one of these, the anterior vocal pathway, is located in the MNM (Jarvis, 2004; Feenders et al., 2008).

In two inactivation studies, a specific region of the MNM, the nidopallium intermedium medialis pars laterale (NIML) had previously been found to contribute to sequential behavior in pigeons (Helduser and Güntürkün, 2012; Helduser et al., 2013). Importantly, these studies suggested a direct interplay between both NIML and NCL in learning and control of behavioral sequences. Two recent studies further demonstrated the importance of NIML in the control of behavior in pigeons. Behroozi et al. (2020) showed an involvement in a go/nogo protocol using high-resolution fMRI. Furthermore, Rook et al. (2021b) showed that NIML is involved in multicomponent behavior using molecular imaging during a stop–change protocol, in which the animals had to observe the presence (or absence) of a stop signal and, accordingly, stop the ongoing behavior, and subsequently change to an alternative behavior. Together, these findings suggest that NIML may be a key component of the avian executive network.

Here we aim to establish, using parallel neurophysiological recordings, to what extent NCL and NIML are involved in (executive) control over sequential behavior. We used a complex and cognitive demanding sequential motor behavior task to investigate the capacity of NIML to mediate motor behavior. Birds were required to keep track of both internally ordered features (i.e., which of two options is valid, based on recent behavioral history) and externally ordered features (i.e., which of two possible sequences is active, based on the outcome of the previous trial) of a sequence (Johnston et al., 2020). The conjunction of both serial order types will allow us to explore how NCL and NIML process sequential information, and to test their involvement and possible differential contribution to executive processing in the avian brain.

## Materials and Methods

### Animals

The experiments were conducted at the Institute of Cognitive Neuroscience at the Ruhr University Bochum. Two experimentally naive pigeons (*Columba livia*), one male and one female animal, were used in the experiment. The animals were obtained from local breeders and housed at the institute animal facility in indoor aviaries or individual home cages. The animals lived under constant room temperature (22°C) with a 12 h light/dark cycle (lights on at 7:00 A.M.; lights off at 7:00 P.M.). All experiments were performed under a food protocol such that the animals were maintained at body weights between 85% and 95% of their free-feeding weight. Animals had free access to water and grit in their home cages. All experimental procedures and housing conditions were conducted in accordance with the National Institutes of Health *Guide for Care and Use of Laboratory Animals* and were authorized by the national authority (North Rhine Westphalian State Agency for Nature, Environment and Consumer Protection).

### Apparatus

Pigeons were trained and tested in an operant chamber measuring 80 cm (height), 54 cm (width), and 56 cm (depth). The chamber was insulated with acoustic foam to reduce ambient noise. Additionally, a ventilator generated constant background noise in the chamber. The operant chamber was further equipped with copper mesh to shield against electromagnetic noise (a Faraday cage) and a surveillance camera. During the testing sessions, the door of the operant chamber was closed, and an LED strip on the ceiling (“house light”) dimly illuminated the chamber. The birds stood on a wooden perch, facing a 54 by 40 cm acoustic pulse touchscreen monitor (Elo Touch Solutions), which displayed the stimuli and detected responses. A custom pellet feeder, which dropped individual pellets (BioServ) into a small feeding trough, delivered food rewards, which were illuminated during the reward period. All aspects of the behavioral task were controlled by a PC using MATLAB (2016a; MathWorks) and the Biopsychology and Psychophysics Toolboxes (Brainard, 1997; Rose et al., 2008). We used a custom ethernet-based digital input/output (I/O) device to control the feeder and lights while also sending event codes to the neurophysiology hardware (code and plans are freely available online at https://www.ngl.psy.ruhr-uni-bochum.de/ngl/shareware/index.html.en).

### Behavioral task and training protocol

**Figure 1.**
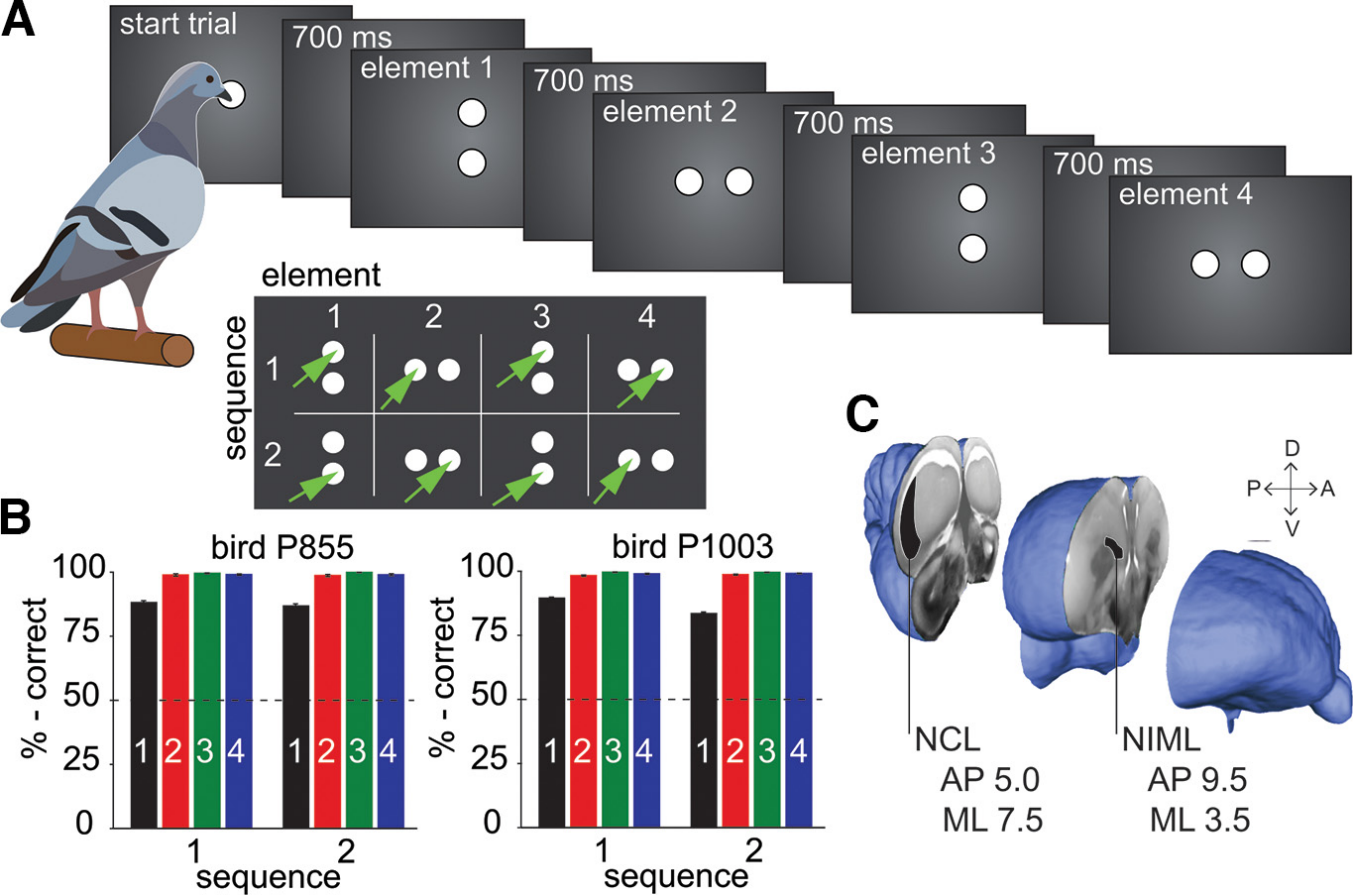
Experimental procedure. ***A***, Behavioral task. Birds performed two different behavioral sequences. Each sequence consisted of four choice elements (at four distinct locations, top, bottom, left, and right). A sequence was repeated until the animal reached a behavioral criterion (plus a pseudorandom number of trials). Sequence transition was not cued and had to be discovered by trial and error. Extended Data Figure 1-1 gives an overview of block lengths. ***B***, Behavioral performance. Both pigeons performed the behavioral task at very high levels, with the lowest performance on the first element of each sequence. Colored bars indicate the mean performance on each element of the behavioral task, for both sequences. Dashed black lines indicate chance level on each element; error bars indicate the standard error of the mean. ***C***, Single-cell activity was recorded in parallel from NCL, and NIML using silicon probes attached to chronically implanted manual microdrives.

The animals were trained to perform two action sequences, presented in a block design. We used a pseudorandom criterion to make the block length unpredictable. The criterion consisted of 16, 20, or 24 correct trials in the current block, with a performance of >80% correct in the last 10 trials. This means that each block consisted of at least 16 trials, but there were also blocks that required at least 20 or 24 trials. During the sessions, the observed block lengths were between 18 and 45 trials (inner 95% of distribution; Extended Data Fig. 1-1). A transition between the sequences was not cued and had to be discovered by trial and error. Thus, the animal had to make an error on the first element of a new sequence block to use the subsequent error signal to switch its behavior. All trials started with a four-second intertrial interval (ITI), after which a white disk (radius, 1 cm) appeared in the center of the screen (Fig. 1). A single peck to this initiation stimulus was followed by a 700 ms delay, after which a sequence of choice elements appeared. These elements consisted of two identical white disks (radius, 1 cm) that were arranged either vertically (first and third elements) or horizontally (second and fourth elements) in the center of the screen. Animals were required to make a single peck to one of the two discs of each choice stimulus. If the animal pecked on the correct location, the two white disks vanished and an interstimulus interval (ISI) of 700 ms passed before the subsequent element appeared. The automated feeder dispensed a food reward following the correct choice on all four elements, which marked the end of the trial. If the animal made an incorrect choice at any time in the sequence, an error signal (2 s white illumination of the screen) appeared, and the trial ended. A recording session consisted of 400 trials in total for bird 1 and of 360 trials for bird 2.

10.1523/ENEURO.0296-22.2023.f1-1 Extended Data Figure 1-1 Distribution of block lengths for sequence 1 and sequence 2. Block lengths had a minimum of 16, 20, or 24 correct trials to ensure that the animals did not learn to anticipate a block change. Download Figure 1-1, TIF file.

To successfully perform this task, the animals must keep track of the current sequence and the element within the sequence. Since all locations were used twice, the correct upcoming location was determined by the active sequence and the ordinal position of the element within the sequence (e.g., Fig. 1*A*, compare element II in sequence 1 to element II in sequence 2). Furthermore, the transition between both sequences had to be inferred from the trial outcome, as no other cue was provided (please refer to the discussion for possible interpretations of the trial outcome as a signal). We first trained the animals in an autoshaping procedure followed by a fixed-ratio (FR) protocol (FR1 followed by FR3) using the initiation stimulus in the middle of the screen. We moved the animals to the next training step when they reached a preset criterion (>70% correct overall performance). Both sequences were trained simultaneously in a “backward” design such that the animals first learned the last (fourth) element of both sequences in a block design. We undertook this approach to encourage behavioral flexibility in switching between the sequences. After the animal reached the criterion, we added the third element of both sequences. In this fashion, we also introduced the second and finally the first element of either sequence. The number of correct trials per block required for a change of sequence was reduced stepwise throughout the training (150, 50, and 30 trials, depending on the proficiency of the animal in the task). Once the animal performed >75% correct on at least two consecutive sessions, it was deemed ready to undergo surgery and to start with data acquisition.

### Surgery

We stereotactically implanted two microdrives in each bird. The electrodes were positioned in NCL [anteroposterior (AP), +5.0; mediolateral (ML), –7.5; dorsoventral (DV), –1.5] and NIML (AP, +9.5; ML, –3.5; DV, –2.3) of the right hemisphere (Karten and Hodos, 1967). Coordinates for the regions were based on histologic studies on the localization of NCL (Waldmann and Güntürkün, 1993; Herold et al., 2011) and NIML (Rehkämper et al., 1985). The birds were anesthetized using isoflurane and received meloxicam (2 mg/kg, i.m.) for analgesia. The skull was exposed, and small craniotomies were made over the target structures. Electrodes and microdrives were fixated with dental acrylic to small bone screws, one of which served as a ground for the recordings. After surgery, the birds received several days of recovery, with monitoring and analgesic treatment of butorphanol (1.5 ml/kg, i.m.). In one of the birds, the left hemisphere was also implanted with the same coordinates in a separate surgery.

### Neurophysiological recordings

Extracellular single neuron recordings were performed using two chronically implanted 32-channel microelectrodes (NeuroNexus). The distance between recording sites was 50 μm for bird 1 (active zone, 1550 μm) and 20 μm (active zone, 640 μm) for bird 2. We recorded both implanted regions simultaneously. Microelectrodes were mounted on movable microdrives (Ddrive, NeuroNexus). The signal was amplified, filtered, and digitized using headstages (model RHD2000, Intan Technologies) and a USB Interface board (Intan Technologies). The system also recorded digital event codes that were sent from the behavioral control PC using a custom I/O device (www.jonasrose.net). Before each recording session, the electrodes were advanced (750 μm) using manual microdrives. Recordings were started 20 min after the advancement, and each electrode was manually checked for neuronal signals. The signals were recorded at a sampling rate of 30 kHz and filtered with a bandpass filter (0.5–7.5 kHz). The recorded neuronal signals were not preselected for task involvement.

We performed spike sorting using the semiautomatic Klusta suite software (Rossant et al., 2016), which uses the high electrode count and their close spacing to isolate signals of single neurons. The spatial distribution of the signal along the different electrodes was considered, by the software, to untangle overlapping signals and separate signals with similar waveform but different recording depth.

### Data analysis

All statistical analyses were performed in MATLAB (2018b; MathWorks) using commercially available toolboxes (Curve Fitting Toolbox, version 3.5.3; Statistics and Machine Learning Toolbox, version 10.2) and custom code. For all statistical tests, we assumed a significance level of α = 0.05. Trials were classified as error trials if, at any element in the sequence, the animal chose an incorrect location. All correct trials were included in the analysis of neural data. For error trial analysis, only trials with an error at the first element (I) were used since the low number of errors on later sequence elements resulted in too few trials for meaningful analysis (Extended Data Fig. 4-1). As a baseline, we chose a 700 ms period of the ITI starting 1000 ms before the start of the next trial.

A neuron was considered task modulated if its firing rate was significantly modulated within a 700 ms period between response to an element and the upcoming element (i.e., in the interstimulus interval). We tested the factors visual *stimulus* (horizontal or vertical alignment of two white disks) and response *location* (Fig. 1*A*, left, right, top, or bottom location of a disk) on the touchscreen with one-way ANOVAs. We tested the factors *sequence* (two possible sequences), *element* in sequence (I–IV), and the interaction between these main factors (*seq*–*ele interaction*) in a two-way ANOVA.

We further calculated a percentage explained variance (PEV) statistic to quantify the effect size of task modulation in the recorded population. Its main parameter ω^2^ (Eq. 1) is a measurement for the percentage to which the tested factor can explain the variance of the data, it is calculated from the sum of squares of the effect (SS_effect_) and the mean squares of the within-group (error) variance (MS_error_). The factors *sequence*, *element*, and *seq*–*ele interaction* were calculated using the partial-ω^2^ measure (Eq. 2), which takes the multifactor design into account, by incorporating the factor-specific mean squares (MS*_f_*) and degrees of freedom (df_effect_), as follows:

(1) $${\text{ω}}^{2}=\frac{{\text{SS}}_{\text{effect}}-\text{df}*{\text{MS}}_{\text{error}}}{{\text{SS}}_{\text{total}}+{\text{MS}}_{\text{error}}},$$

(2) $${\text{ω}}_{p}^{2}=\frac{{\text{SS}}_{\text{effect}}-{\text{df}}_{\text{effect}}*{\text{MS}}_{f}}{{\text{SS}}_{\text{effect}}+\left(N-{\text{df}}_{\text{effect}}\right)*{\text{MS}}_{f}}.$$

The population PEV analysis was conducted on the time interval starting 700 ms before the response to an element until 700 ms after the response, with a bin size of 100 ms and a step size of 10 ms. We calculated a permutation test to check whether the calculated values of effect size were significant. We ran the permutation to calculate the likelihood of getting an explained variance value larger than the one calculated from the actual distribution of the data by permuting the dataset randomly and calculating the PEV 1000 times. The test thereby does not assume any distribution of the data and returns an unbiased estimate of the likelihood of generating an effect size within the data randomly. The measured value of explained variance from the actual dataset was assumed to be significant if the likelihood of randomly generating a larger value was <5%.

To test whether the neural response to *reward* differed from baseline activity a one-way ANOVA was used to compare the response during the reward period of correct trials with an equally long period (700 ms) in the middle of the ITI. For analyses directly comparing correct and error trials, we only used trials in which an animal made an incorrect choice for the first sequential element, because the number of errors for the other sequence elements (i.e., elements II, III, and IV) was too low for analysis. Here we calculated a two-way ANOVA with the main factors *sequence* and *outcome* (i.e., reward vs error feedback) and *seq*–*outcm interaction*. Because there were substantially fewer error trials than correct trials, we subsampled correct trials with the amount of error trials and performed the ANOVA on these trial-equalized groups. For the error trial analysis on the population level, the interval starting 1000 ms before the erroneous peck up to the initiation stimulus of the next trial (i.e., 5000 ms after the error) was used (bin size, 100 ms; step size, 100 ms).

Neuronal activity of behavioral switches between the sequences was analyzed using a two-way ANOVA with the main factors *sequence* and *switch* (i.e., trial had behavioral switch, vs trial did not have a behavioral switch), and the *seq*–*switch interaction*. Here the analysis period was aligned relative to the response to the initiation stimulus beginning 3000 ms before the response (during ITI) and ending 1000 ms after the response (bin size, 10 ms; advanced with a step size of 10 ms). Only trials in which a bird performed a successful switch (i.e., the first correct trial of the new sequence) were analyzed. We equalized the sample sizes of switch trials and nonswitch trials by randomly subsampling nonswitch trials with the amount of switch trials. Information about the upcoming switch was derived from the effect size ( ${\text{ω}}_{p}^{2}$) of the factor switch. We determined whether a neuron had significant information about the switch by applying the permutation test introduced above to the PEV of each neuron in the interval ±1 s around the initiation response.

### Data availability

The data and code underlying the results reported in this article is available under the following DOI: 10.5281/zenodo.6044274.

## Results

### Behavioral performance

We trained two pigeons on an action sequence protocol. Each sequence consisted of four choices (Fig. 1*A*). Within each session, the animals had to switch multiple times between two sequences, relying only on error feedback to initiate the switch. The overall behavioral performance of both birds was very high (Fig. 1*B*) and well above chance for all elements in both sequences (binomial test, all *p* < 0.001). Both birds had a performance of close to 100% for the elements II–IV regardless of sequence (range, 98.67–98.89%). The performance for element I was significantly lower compared with the other elements, for both birds and both sequences (effect of element: bird 1, seq1: *F*_(3,56)_ = 127, *p* < 0.001, ω^2^ = 0.86; bird 1, seq 2: *F*_(3,56)_ = 113, *p* < 0.001, ω^2^ = 0.84; bird 2 seq 1: *F*_(3,152)_ = 282, *p* < 0.001, ω^2^ = 0.85; bird 2 seq 2: *F*_(3,152)_ = 441, *p* < 0.001, ω^2^ = 0.89; *post hoc* Tukey–Kramer test: both birds, both sequences: all comparisons to element I, *p* < 0.001; all other comparisons, *p* > 0.05; exception bird 2, sequence 1: comparison of element II to element III, *p* < 0.001). Performance of both birds in sequences 1 and 2 did not differ significantly for elements II–IV (repeated-measures ANOVA: *F*_(1,43)_ = 0.23, *p *=* *0.64, ω*^2^* = −0.01; and *F*_(1,115)_ = 1.44, *p *=* *0.23, ω*^2^* = −0.0003). A separate dependent *t* test was calculated for element I. For bird 1, there was no significant difference between the sequences (*t*_(14)_ = 1.33, *p* = 0.21, *d* = 0.52); for bird 2, there was a significant difference between the two sequences (*t*_(38)_ = 6.26, *p* < 0.001, *d* = 1.49). Following a block transition (i.e., when the response to the first element of the previously correct sequence suddenly produced error feedback), both birds performed a switch from one motor sequence to the other quickly, within averages of 2.77 (±0.20; bird 1) and 3.33 (±0.20; bird 2) trials. In contrast, when the birds produced errors that occurred within blocks (i.e., when the bird started with the incorrect element or incorrectly transitioned between elements), they usually returned to the correct sequence in the following trial. The average number of additional incorrect trials following such a within-block error was only 0.43 (±0.05; pigeon 1) and 0.52 (±0.03; pigeon 2). This indicates that the birds were tracking their behavioral outcome and knew how to adapt behavior when they received error feedback (please refer to the Discussion for possible interpretations of the outcome as a signal to guide behavior).

### Sequence information is represented in NCL and in NIML

**Figure 2.**
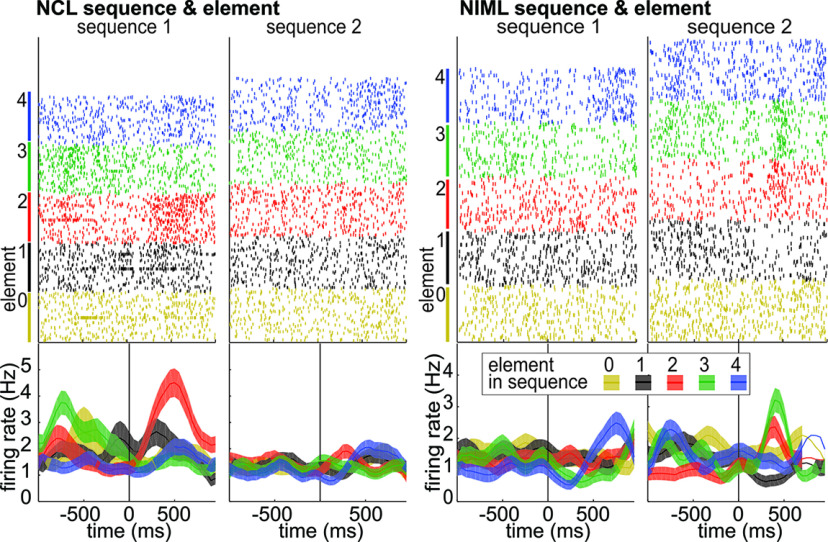
Neurons represent the interaction of sequence and element. Raster plots (each dot represents one action potential) and histograms (average firing rate and standard error of the mean) of example neurons recorded in NCL and NIML. Only correct trials are depicted. Both neurons responded stronger to particular elements in only one of the two sequences. For example, the NCL neuron responds with a substantial increase of firing rate to element II (red), but only for sequence 1. This neuronal activity is best explained by the interaction of element and sequence. Alternative explanations like the target location do not fit the observed differences. Consider the red curve of sequence 1 and the blue curve of sequence 2; despite the same location (left) being the target, the firing rates differ substantially. Colors indicate neural response to the start stimulus (yellow), and elements I (black), II (red), III (green), and IV (blue). Data are aligned to the response to each element (time 0/black vertical line). In the raster plot, each trial is depicted four times (once for each element).

During task execution, we simultaneously recorded single-neuron responses from NCL (110 neurons) and NIML (152 neurons). In one bird, we recorded from both the left and the right hemisphere consecutively. We did not observe any difference between hemispheres. Recordings were performed in a total of 55 sessions, each contributing 360–400 trials. We tested whether the recorded neurons were modulated by nonsequential task-relevant aspects by performing one-way ANOVAs with the factors *visual stimulus*, *response location*, and *reward*. The sequential factors *element*, *sequence*, and *seq*–*ele interaction* were tested with two-way ANOVA. For instance, the response of a neuron was classified as modulated by *seq*–*ele interaction* if a two-way ANOVA revealed a significant interaction of the main factors sequence and element in the ISI among the four elements and between the two sequences. An example neuron from NCL and NIML, significantly modulated by *seq*–*ele interaction*, can be found in Figure 2.

Overall, we found that individual neurons were commonly modulated by more than just one of these aspects of the task (Extended Data Fig. 3-1). The relative numbers of task-modulated neurons were comparable between the two regions and a χ^2^ test did not reveal a difference between these numbers (all ${\text{χ}}_{\left(1\right)}^{2}$, <3.3; all *p* > 0.05). Within the two regions, neuronal populations that represented sequential information were not clearly separated. Most neurons responded to combinations of the factors *element*, *sequence*, and *seq*–*ele interaction* (NCL, 50.00%; NIML, 61.18%; Extended Data Fig. 3-1, “interaction & any main”; i.e., the firing rate of these neurons was significantly affected by any two combinations the factors). Furthermore, there was an overlap between the representation of the sequential factors (*sequence* and *element*) and nonsequential factors (*response location* and *visual stimulus*), both modulating neuronal activity of many neurons. However, a substantial fraction of all neurons (NCL, 17.28%; NIML, 15.79%) showed significant modulation for at least one of the sequential factors (*sequence*, *element*, *seq*–*ele interaction*), without being modulated by nonsequential factors (*location* or *stimulus*; Extended Data Fig. 3-1, bottom row).

**Figure 3.**
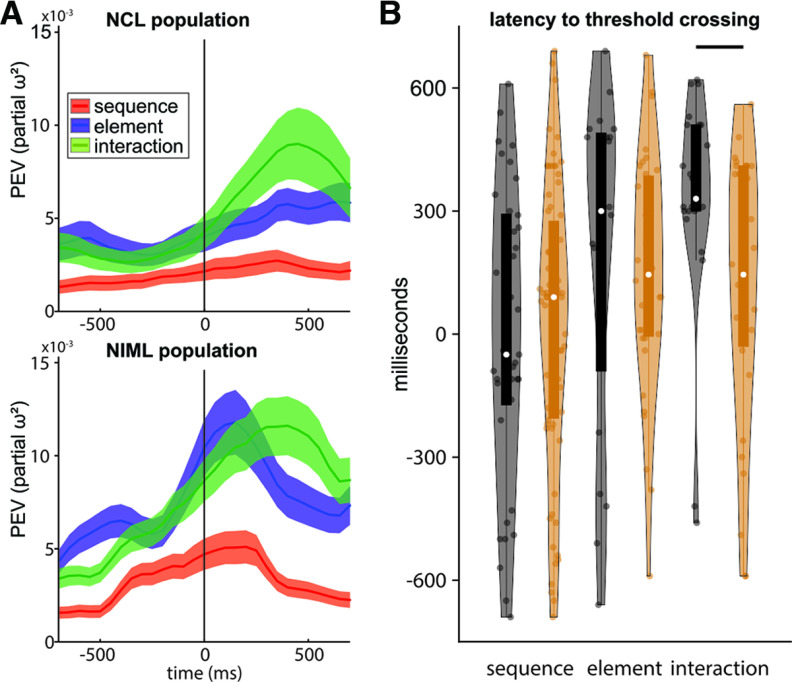
NCL and NIML have similar response profiles but differ in timing. ***A***, Population response in NCL (top) and NIML (bottom). Sequential information expressed as PEV by *sequence* (red), *element* (blue), and *seq*–*ele interaction* (green). The vertical black line indicates the time of response. Both regions represented elements and interactions, while sequence was represented mostly in NIML. Importantly, information about *seq*–*ele interaction* emerges earlier in NIML than in NCL. Lines indicate the mean; shaded area indicates the standard error of the mean; PEV is across all recorded neurons. ***B***, The interaction emerges in NIML (orange) significantly earlier (indicated by horizontal black line) than in NCL (black). Violin plots of latencies of the two regions measured as the first bin crossing the information threshold (3 standard deviations above the mean information of a neuron; only neurons crossing the +3 standard deviations level were taken into account; based on 100 ms bins advanced with a step size of 10 ms). Extended Data Figure 3-1 gives an overview of significant neuron counts in both regions for the different test groups.

10.1523/ENEURO.0296-22.2023.f3-1 Extended Data Figure 3-1 Percentage of significant neurons by factor and different factor combinations. Cells indicate the proportions of significant neurons. There was one two-way ANOVA (*sequence*, and *element*, *seq*–*ele interaction*) and four one-way ANOVAs (*visual stimulus*, *response location*, *outcome*, respectively). The bottom most row denotes the percentage of neurons significant for the *seq*–*ele interaction* while having no significance for either *visual stimulus* or *response location*. Download Figure 3-1, DOC file.

### Latency of sequential information

To compare the processing of task-relevant information between NCL and NIML, we calculated PEV (partial ω^2^; for details, see Materials and Methods) in the entire recorded population. Both regions encoded *seq*–*ele interaction* in between responses to the individual elements (Fig. 3*A*). This indicates that both regions encoded the critical aspects of the ongoing sequential task and could be involved in determining the correct choice to a particular visual stimulus, as a function of where the animal was in the ongoing sequence.

We next calculated the latency difference between the regions to investigate their temporal relationship. Looking at the information about *sequence*, *element*, and *seq*–*ele interaction* on the single-neuron level showed that there was a substantial overlap of neuronal activity between the two regions. Therefore, to quantify the difference, only neurons crossing a threshold of 3 SDs above their mean PEV value were considered (mean baseline interval, ±700 ms around response; 100 ms bins; and 10 ms step size). We defined the first time this threshold was crossed as the latency of the neuron. The temporal distribution of incidences of these events was similar for the main factors *sequence* and *element*, neither of which showed a significant difference between the two regions (*z* = −0.32, *p* = 0.75, *d *=* *0.08; and *z *=* *1.09, *p *=* *0.27, *d *=* *0.09; Fig. 3*B*). The latency for the *seq*–*ele interaction*, however, did show a clear separation of the two regions, with NIML showing the specific modulation significantly earlier than NCL (*z *=* *2.54, *p *=* *0.0109, *d *=* *0.72; Fig. 3*B*).

### Encoding of behavioral outcome and sequence-switch

**Figure 4.**
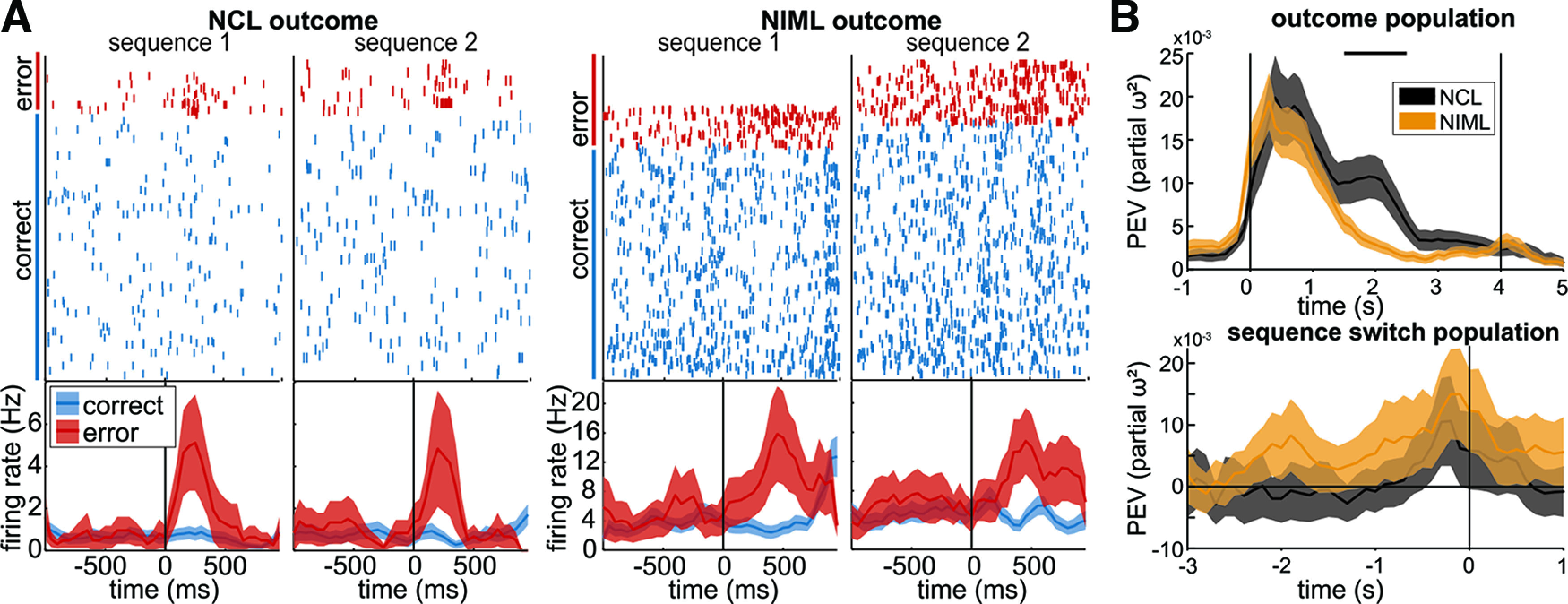
Choice outcome and sequence switch in NCL and NIML. ***A***, Raster plots and histograms of example neurons in NCL (left panel) and NIML (right panel). Correct and error trials for element I are depicted. Both neurons responded strongly to erroneous responses. Colors indicate the neural response to element I (correct, blue; incorrect, red). Data were aligned to the response (time 0/black vertical line). In the raster plot, correct and incorrect trials are grouped. ***B*** (top) Population response to the outcome (element IV correct vs element I incorrect) in NCL (black) and NIML (orange). Both regions differentiate between correct and error trials, but NCL does so slightly stronger and for a longer period. Lines indicate the mean and shaded areas indicate the standard error of the mean of PEV across all recorded neurons. Vertical black lines indicate the time of response (0 s) and the end of the feedback phase (4 s), and horizontal black lines indicate a significant difference between the regions. See Extended Data Figure 4-1 for the number of error trials per element and statistical values, and Extended Data Figure 4-2 for statistical values and results concerning the factor outcome. ***B*** (bottom) Subpopulation PEV sequence change (switch vs no-switch) in NCL (black) and NIML (orange). Only correct sequence switches, when the animals successfully transitioned from one sequence to the other, and all other correct trials, where the animals perform the same sequence from trial to trial, are depicted. Both regions differentiated switch from no-switch trials before the onset of the next sequence. Vertical black lines indicate the response to the initiation stimulus (0 s). Lines indicate the mean, and shaded areas the standard error of the mean of PEV across all selected neurons. Horizontal black lines indicate significant differences between the regions. See Extended Data Figures 4-3, 4-4, 4-5, 4-6 for statistical values and results concerning the factors *sequence*, *switch*, and *seq*–*switch interaction*.

We used two different approaches to investigate a representation of trial outcome. First, we analyzed reward modulation by comparing neuronal baseline activity to activity following a correct response to the last element (element IV) of both sequences using a one-way ANOVA. A large fraction of neurons in both regions (NCL, 60.00%; NIML, 59.21%) were reward modulated according to this criterion. The following error trial analyses were based only on errors on the first element of either sequence since too few errors were made on the following elements (see Materials and Methods, Data analysis; Extended Data Fig. 4-1). Many neurons of both regions responded differently following correct and incorrect choices (NCL, 40.00%; NIML, 38.16%; Fig. 4*A*).

10.1523/ENEURO.0296-22.2023.f4-1 Extended Data Figure 4-1 Overview of error trials. Average number of errors and percentage of errors of all trials for sequences 1 and 2, sorted by the element at which an error occurred. Values in parentheses indicate the SEM (means calculated across sessions). Download Figure 4-1, DOC file.

10.1523/ENEURO.0296-22.2023.f4-2 Extended Data Figure 4-2 Statistical results of *t* test for outcome PEV. Download Figure 4-2, DOC file.

10.1523/ENEURO.0296-22.2023.f4-3 Extended Data Figure 4-3 NCL and NIML populations during switch trials. Population PEV for the factors sequence, switch, and their interaction in NCL (black) and NIML (orange). Only correct sequence switches, when the animals successfully transitioned from one sequence to the other, and all other correct trials, where the animals perform the same sequence from trial to trial, are depicted. NIML differentiated switch from no-switch trials (orange bar at the top of factor switch before the onset of the next sequence. Vertical red lines indicate the response to the initiation stimulus (0 s). Lines indicate the mean and shaded areas the SEM of PEV across all neurons. Download Figure 4-3, TIF file.

10.1523/ENEURO.0296-22.2023.f4-4 Extended Data Figure 4-4 Significant time bins (in the interval –2000 to 1000 ms relative to sequence initiation) based on results of the permutation for switch (1000 permutations; significant if probability was <2.5%) for the whole population. Bin width, 100 ms. Download Figure 4-4, DOC file.

10.1523/ENEURO.0296-22.2023.f4-5 Extended Data Figure 4-5 Significant time bins (in the interval –2000 to 1000 ms relative to sequence initiation) based on the results of the permutation for switch (1000 permutations; significant if the probability was <2.5%) for the subpopulation. Bin width, 100 ms. Download Figure 4-5, DOC file.

10.1523/ENEURO.0296-22.2023.f4-6 Extended Data Figure 4-6 Statistical results of *t* test for factor switch PEV between NCL and NIML subpopulations. Download Figure 4-6, DOC file.

We further analyzed the effect of trial outcome at the neuronal population level with a two-way ANOVA with the main factors *sequence* and *outcome*. We did this by comparing a correct response to element IV with subsequent reward to an incorrect response to element I with the subsequent error signal. Both regions showed modulation after the response, differentiating between error and correct trials, but the average PEV for the *outcome* was significantly higher in NCL than in NIML (all *t*_(260)_ > 2.07, all *p *<* *0.05, all *d *>* *0.24, two consecutive nonoverlapping bins were significant; Fig. 4*B*, Extended Data Fig. 4-2).

Following an error, the bird had to switch the executed sequence, whereas following a correct trial, the bird could stay with the current sequence. We analyzed the information about this sequence switch by calculating the PEV with a two-way ANOVA, with the factors *sequence* and *switch*, and quantifying the effect size (see Materials and Methods, Data analysis). A subpopulation of neurons in both regions (NCL, 16.36%; NIML, 15.13%) reached significant information (PEV) about the switch, but not about the sequence identity, or the sequence–switch interaction (Extended Data Fig. 4-3). Significance was reached first in NIML (−300 to 100 ms before initialization) and later in NCL (−200 to 0 ms before initialization; Fig. 4*C*, Extended Data Fig. 4-4). The amount of information about the switch, encoded by the two subpopulations of NCL and NIML, did not show a significant difference. This indicates that both regions had information about the upcoming switch of sequences before the onset of the sequence.

## Discussion

We investigated the neural correlates of control and execution of behavioral sequences in pigeons. The birds had to attend a series of visual stimuli to select a response based on knowledge about the current sequence and about the current element within the sequence. The pigeons further had to detect a transition between two sequences based on the outcome (i.e., error–feedback, but see the Discussion below about this observation) following an incorrect response. Therefore, the animals had to make an error to know that a switch had occurred. Using this feedback, the animals quickly adapted their choices within a few trials, indicating that the birds were able to flexibly transition between the sequences based on feedback.

### The NCL processes sequence information and keeps track of the current action plan

A large proportion of the neurons recorded from NCL responded to the presented *visual stimulus*, the *interaction*, and/or main effects of *sequence* and *element*, the *response location*, and/or the *outcome* of the trial, thereby encompassing all information required to perform in the behavioral task. Some of these factors are well established for NCL.

Neurons in NCL are selective for behaviorally relevant visual stimuli (Veit and Nieder, 2013), target locations (Veit et al., 2015), and trial outcomes (Kalt et al., 1999; Browning et al., 2011; Starosta et al., 2013; Veit et al., 2017). In contrast to the rich data showing representations of nonsequential stimuli in NCL, there is less neurophysiological evidence for sequential information. Nonetheless, the involvement of NCL in sequential behavior is well established. NCL projects to downstream motor regions (Kröner and Güntürkün, 1999; Güntürkün and Bugnyar, 2016), and its inactivation induces transition errors in a motor sequence (Helduser et al., 2013). However, the impairments following NCL inactivation might not necessarily be specific to sequential behavior. Johnston et al. (2018) found that the impairments caused by TTX injections into NCL were comparable between a serial-order protocol and a simple go/nogo protocol without sequencing requirements arguing for a general role of NCL that is not specific to ordering/sequencing behavioral tasks. Further, a neurophysiological study in NCL compared internally ordered (the animal can choose freely as long as it does not respond to a previously chosen stimulus) and externally ordered (the animal must choose in a specific sequence) serial-order protocols (Johnston et al., 2020). The authors found ordinal stimulus representations in the internally, but not the externally, ordered protocol supporting the notion that NCL is involved in sequential behavior but is very likely not the only structure involved—possibly depending on minutia of the behavioral task. Our data support this and provide evidence that NCL represents all of the information required to perform in a behavioral task that requires executing and switching between two sequences.

### Neuronal activity in NIML shows characteristic signatures of being a higher executive region

To our knowledge, the present study is the first to investigate single-cell activity of NIML and therefore is the first to report that these correlates in NIML are remarkably similar to those of NCL. All of the sequential and nonsequential factors influencing the activity of neurons in NCL (mentioned above) also affected neuronal activity in NIML. Hence, NIML equally represents all relevant information required to perform the behavioral sequences. This is in line with studies reporting that immediate early genes are upregulated in NIML after movement (Feenders et al., 2008) and multicomponent behavior (Rook et al., 2021b), and fMRI results correlated with multicomponent behavior (Behroozi et al., 2020). This redundancy of information between NCL and NIML seems puzzling but may offer an exciting possibility: NIML may be an additional region for executive functions. However, there are three observations that require clarification before further considering NIML in such a context.

First, as was previously reported for NCL, most selective neurons of NIML were modulated by multiple task components. Therefore, it might be possible that a strong neural representation of nonsequential factors gives rise to a false positive in the analysis of sequential factors. For instance, a neuron could strongly represent only one target location, and this activity could be interpreted as interaction between *sequence* and *element*. However, this explanation is unlikely since many neurons were significant for sequential factors (*sequence*, *element*, or *interaction)* but did not respond to the critical nonsequential factors (*stimulus* or *location*). Additionally, the comparatively large effect sizes for *sequence*, *element*, and *interaction* at the population level make this interpretation unlikely—even for the neurons that responded to multiple factors. A more parsimonious explanation is therefore that neurons in both regions showed “mixed selectivity,” as was previously reported in associative structures such as the mammalian PFC (Rigotti et al., 2013) and the avian NCL (Moll and Nieder, 2017).

Second, neuronal activity in NIML might only relay information from NCL without being directly relevant for behavioral output. By recording simultaneously from NCL and NIML, we were able to compare the time course of neural signals between these structures. We found that information about the current sequential element (i.e., *seq*–*ele interaction*) first appeared in NIML and reached NCL only after a delay of 50–300 ms (Fig. 3). This latency could indicate that NCL activity is driven by NIML and consecutively drives motor output. However, a critical role of NCL in relaying motor output from NIML seems unlikely because the inactivation of NCL did not fully abolish sequential behavior in previous studies (Helduser and Güntürkün, 2012; Helduser et al., 2013; Johnston et al., 2018). Instead, feedback about the current status of an ongoing behavior (i.e., *seq–ele interaction*) might reach NCL via NIML. This would fit the likely role of NCL to guide behavioral flexibility, for example, by integrating motor output with multimodal feedback. This is a requirement for the successful selection of upcoming elements and for switching between the two sequences, as feedback is required for the adaptation and guidance of motor commands. It is also consistent with results from the inactivation study of Helduser et al. (2013), where element transitions became more erroneous following both NCL and NIML inactivation.

Finally, if NIML were to be a higher executive region, it should in some way show signs of integration of information to guide behavior. For example, this is the case for NCL, for which a role in behavioral flexibility was previously reported using lesions and receptor blockade that resulted in deficits in a reversal task (Hartmann and Güntürkün, 1998; Lissek et al., 2002). Here we found evidence of integration of information not only in NCL but also in NIML. This can be seen at the level of individual neurons with specific tuning to the rewarded outcome, and from the differential response of the population to the trial outcome.

We interpret the neural correlates of the trial outcome (white feedback stimulus and reward), arising from differential firing rates, as abstract feedback signals containing information about the outcome. It is already well established that NCL plays a role in the feedback evaluation. However, it is important to note that we cannot distinguish with certainty whether the neural responses reported here are indeed abstract feedback signals or mere representations of the physical cues we provided: reward delivery or a screen flash on error trials. In the absence of a definitive test of the neuronal activity, we consider the following two lines of reasoning for our interpretation. (1) Neuronal activity of NCL has been found to have an abstract quality (i.e., independent of physical stimulus properties). For example, neurons of NCL encode rules (Veit and Nieder, 2013), numerosity (Ditz and Nieder, 2015; Kobylkov et al., 2022), and stimulus category (Kirsch et al., 2009; Anderson et al., 2020). Furthermore (2), reward strongly affects neuronal activity in NCL in the form of reward magnitude (Koenen et al., 2013), availability (Rose and Colombo, 2005; Johnston et al., 2017), and proximity (Kalenscher et al., 2005; Scarf et al., 2011). Importantly, it was shown that NCL encodes the reward prediction error, a key feedback signal in learning theory (Starosta et al., 2013; Puig et al., 2014; Packheiser et al., 2021). Since this is the first study recording single cells in NIML, there is no direct evidence in the literature that demonstrates neural correlates of feedback processing. However, much like NCL, NIML is not a sensory visual area. Additionally, NIML inactivation increases sequence errors (Helduser and Güntürkün, 2012; Helduser et al., 2013), and immediate early gene expression is increased when the animals stop ongoing behavior (Rook et al., 2021b). Both results were interpreted as evidence for a role of NIML in abstract processes. Importantly, we found that the response to feedback stimuli occurred later in NIML than in NCL. The latter is commonly regarded as the highest associative structure in the avian brain. Thus, low-level sensory processing of highly familiar feedback stimuli in NIML after the feedback response in NCL seems rather unlikely. Together the neural correlates reported here can be interpreted in line with abstract feedback signals, especially in NIML; however, additional experiments will be required to put this interpretation to the test.

Outcome monitoring is crucial to flexibly switch behavior after an erroneous response, in this instance to switch from executing one sequence to executing the other. The neural representation of trial outcome was initially present in both regions but declined faster in NIML than in NCL, which maintained the information about the outcome almost to the end of the feedback phase. The outcome was not sustained throughout the ITI in either region. This may indicate that the switch between the sequences was initiated only at some point shortly before the onset of the next trial, instead of being maintained following the outcome of the previous trial. Indeed, shortly before the onset of the subsequent trial, the population activity of both regions differentiated between trials with an upcoming sequence switch and the continuation of the same sequence. This suggests that both NCL and NIML played a role in the process of switching sequences. Overall, the two regions encoded the switch very similar, peaking at virtually the same time. The only difference we detected was some NIML neurons that showed the differentiation earlier than NCL.

### The NCL and NIML of pigeons may work in concert as a system for higher executive functions

We found neural correlates of all relevant task components in NCL, and importantly, also in NIML. This suggests that these structures might work together as part of an executive network controlling sequential behavior. Our data were not conclusive about direct network interactions such as neurons from one region producing direct input at neurons of the other region or any correlation between instances of spiking. However, our data suggest that between the two regions the role of NCL is evaluative of, rather than instructive for, motor-related signals. NCL processes sequential information after NIML (as indicated by the higher latency relative to a peck), and it has a more pronounced response to errors than NIML. Therefore, we suggest that NIML might relay information related to sequential motor output to NCL, where it can be integrated with multimodal sensory feedback signals (auditory, visual, and tactile). The integrated signal can then be projected to the arcopallium (the motor output region), where it may converge with the direct projections from NIML (Zeier and Karten, 1971; Kröner and Güntürkün, 1999). Inactivation of parts of this anatomic network result in transition errors and more general deficits in motor sequence production (Helduser et al., 2013; Johnston et al., 2018). This might be because of a lack of feedback integration of the ongoing sequence (e.g., body posture, beak position, and visual and auditory cues) that would affect the identification of the appropriate upcoming sequential step, or (agnostic to specific functions) because the network dynamics of a downstream motor region are perturbed by the transient inactivation of an upstream signal source (Otchy et al., 2015). To disentangle these possibilities, recordings in arcopallium, in combination with transient and permanent inactivation of NIML and NCL will be required. The recent development of optogenetic methods in pigeons (Rook et al., 2021a) makes this a possibility. Importantly, the suggested functions still require experimental cross-validations, with neuronal recordings of the respective regions in different tasks to test specific contributions. The role of NCL in this network should be considered equivalent to PFC, based on a wealth of neurophysiological data (Nieder, 2017). Because of the lack of such data, the role of NIML is naturally much more difficult to grasp. We observed the termination of specific behavior and a subsequent change to an alternative behavior, the sequential switch. It has been suggested that this is reminiscent of functions of right inferior frontal gyrus in humans, while multimodal innervation of the MNM and its potential role in movement preparation is similar to mammalian posterior parietal cortex (Rook et al., 2021b). Irrespective of the exact relationship of NIML to mammalian structures, our results indicate the presence of more than one avian executive region with both NCL and NIML playing a crucial role in feedback-based behavioral flexibility. To further lay a foundation of an avian executive network, it will be crucial to directly measure whether and how information from one region is transmitted to the other.

## Synthesis

Reviewing Editor: Christine Portfors, Washington State University Decisions are customarily a result of the Reviewing Editor and the peer reviewers coming together and discussing their recommendations until a consensus is reached. When revisions are invited, a fact-based synthesis statement explaining their decision and outlining what is needed to prepare a revision will be listed below. The following reviewer(s) agreed to reveal their identity: Dmitry Kobylkov.

The reviewers agree that this manuscript provides interesting and timely data addressing neural encoding sequence behavior in the avian forebrain. This is an understudied topic and this manuscript provides an important step in understanding who different forebrain regions contribute to higher cognitive functions. The authors agree however that the authors need to more clearly determine whether the neural activity reflects processing sequence or the processing of location. The reviewers are not convinced that the results demonstrate sequential processing.

Specific comments:

Methods:

1. Line 131: Do I understand it correctly that you added some trials to make the block length unpredictable? How many did you add? 16, 20 or 24 correct trials out of how many trials? And why exactly this amount of correct trials, does it correspond to 80%?

2. Line 163: How many trials are in one block?

3. Line 176: Was there any difference between hemispheres? You don’t mention it in the results.

4. Line 205: How many error trials occurred during the elements 2-4 compared to the element 1?

4. Line 213: since the stimuli within one sequence are interconnected, i.e. the response to the first element predicts responses to subsequent elements, shouldn’t we expect that the neural responses are also not independent within one trail. Then, strictly speaking, treating them as independent in the ANOVA would be not correct.

Results:

If I understand it correctly, there are two possibilities when an error could occur at the element 1: if the sequence has been changed and the first element should be used by the bird as a cue so they are deemed to make an error; if the sequence has not been changed and the error does not indicate the sequence switch. You analyse only the first type of errors, it I get it correctly. Was there any difference at the neural/behavioural level between these two types of errors?

Figure 2: In the example of the NIML neuron from the figure 2 it is not clear, what element of the sequence this particular neuron is responding to (it might be element 3, or both 2 and 3). Did you perform post-hoc analysis to quantify how many of your seq-ele neurons actually code for one specific element?

Line 323: by “combinations of the factors” do you mean that their firing rate depends on at least one of the factors or an interaction of them?

Figure 3: The amount of neurons that potentially encode the sequence is very low (<5% ?). Is it still above the false positive rate? Maybe you could create a table with ANOVA results and the number of corresponding neurons for each category? Or at least add the description of single bar graphs into the legend, because it is rather confusing now. For example, are neurons from “only element” group also included in the “only seq & elem” group as well?

Figure 4: After an error the white screen appeared for 2 seconds. How do you know that the neural response to the error is not the response to the visual stimulus (white screen)? The example neuron in NIML has a strong response at the end of the analysed period in the correct trials of the first sequence. Where does this response come from?

Discussion:

Lines 480-482: While authors did such an effort to record simultaneously from two forebrain regions, I found it rather disappointing that the only analysis they provide is the latency of neural responses. To compare the latencies no simultaneous recording is needed. Instead, it would be great to see some cross-correlation analyses to maybe identify neurons that get direct input from NIML to NCL. I assume that authors actually did perform this analysis, but such direct neuron-to-neuron responses are obviously very challenging to find. So if this is the case, I would suggest that authors share this part of the analysis as well. It wouldn’t diminish the quality of their study, but actually help other people to appreciate the difficulty of the method. Therefore, I do not fully agree with the title which includes words “executive network”, because authors did neither provide a network analysis, nor did they analyse the full network.

Line 75: as a “stop-change protocol” may not be a very familiar procedure (it was not to me), possible expand with a few more words

Line 77: did you mean “event” or “extent”

Line 114: change “ventilating fan-generated” to “ventilate fan generated”

Line 210: you tested the factor of response location. I was a bit unsure what “location” refers to. Do you mean left or right in the horizontal display, and up or down in the vertical display?

Line 314-315: You have a cell that fires to the second element of sequence 1 and that it can’t be explained by reward proximity, stimulus, or response location. The second element of sequence 1 is a response to the left on a horizontal display. The response to the left of the horizontal display also occurs in the fourth element of sequence 2. When I look at Figure 2 on the left it definitely fires to the fourth element of sequence 2, so to me this is not a cell that codes sequential information but rather a cell that simply fires to the left location on the horizontal display. It is very clear from the figure. How do you counter that argument?

Similarly, the NIML cell in Figure 2 fires to the third element of sequence 2 which is the same position as the first element of sequence 2, and it certainly fires to the first element of sequence 2 as well. True, it does so in an inhibitory manner, but that is a response nonetheless. Again, to me this is a cell that fires to the bottom location of a vertical display. And as you noted in Figure 3a, most of your cells are indeed location cells.

Figure 3a: To me, the number of cells that yielded a significant factor of sequence, or sequence and element, are around 5%. Given the large number of tests you conducted, would you not expect about 5% to come out significant that weren’t?

Figure 3b: On my computer, the element legend is blue, but the element graph is purple.

To me, your data are clear that there are no difference between NCL and NIML in terms of the response profiles. The only difference is a slightly earlier, in the region of around 200 msec, emergence of a neural response in NIML, and the fact that the PEV for outcome was higher in NCL than NIML. Two comments.

1. One comment is whether what we are dealing with raises the issue of statistically significant vs statistically meaningful. In brain terms 200 msec is quite long, I agree, but I wonder whether connections/anatomy can explain the difference, rather than differences in some form of sequential processing.

2. Second, a complex story is created around this difference in terms of what the NCL and NIML might be contributing to sequential behaviour. Given the similarities between the areas on so many other dimensions, is there no other, simpler, interpretation of the contribution of these regions to sequential behaviour other than outcome monitoring? To me the fact that you have identified another potential executive area in the avian brain is good enough.

## Author Response

Synthesis of Reviews:

Computational Neuroscience Model Code Accessibility Comments for Author (Required):

N/A

Synthesis Statement for Author (Required):

The reviewers agree that this manuscript provides interesting and timely data addressing neural encoding sequence behavior in the avian forebrain. This is an understudied topic and this manuscript provides an important step in understanding who different forebrain regions contribute to higher cognitive functions. The authors agree however that the authors need to more clearly determine whether the neural activity reflects processing sequence or the processing of location. The reviewers are not convinced that the results demonstrate sequential processing.

We hope that our detailed answers below will help to clarify why we think that the neuronal activity in both NCL and NIML reflects sequential processing (in a precise sequence-element fashion). We think that the changes we have made to the manuscript will highlight that the neuronal activity in both regions can best be explained by such specific interactions and that processing of element locations does not offer a satisfying alternative explanation. Please refer to our responses below for more detailed accounts on specific issues.

Specific comments:

R1---------------------------------------------------------------------------------------------------------------------------------------

Methods:

1. Line 131: Do I understand it correctly that you added some trials to make the block length unpredictable? How many did you add? 16, 20 or 24 correct trials out of how many trials? And why exactly this amount of correct trials, does it correspond to 80%?

Block length depended on two ciriteria to keep it unpredictable but also dependent on the birds’ performance. To transition from one block to the next we randomly required at least 16, 20 or 24 correct trials with 80% correct on the last 10 trials. We used the randomization to ensure that the birds were not anticipating an upcoming block change, but instead relied on the provided feedback following the peck on element 1. The empirically observed block lengths (see next question) indicate that this procedure was successful. We have added this clarification to the manuscript (lines 129 ff.)

2. Line 163: How many trials are in one block?

Block lengths varied based on randomization and performance. We have added an overview of the range of block lengths that appeared throughout recording. Overall, block lengths were between 18 and 45 trials (inner 95 % of distribution, Review Fig. 1/ Extended Data Fig. 1-1), trial number per block rarely exceeded 50.

Review Figure 1: Proportions of specific block lengths for both birds and sequences 1 and 2. Most observed block lengths fell between 18 and 45 trials.

3. Line 176: Was there any difference between hemispheres? You don’t mention it in the results.

We did not observe any difference between hemispheres. We have clarified this in the results section (lines 303 f.).

4. Line 205: How many error trials occurred during the elements 2-4 compared to the element 1?

The number of error trials was very low, in particular for elements 2-4, we have added the following table to the manuscript to give an overview (Review Table 1/Extended Data Figure 4-1).

Review Table 1: Overview of error trials. Average number of errors and percentage of errors of all trials for sequences 1 and 2, sorted by the element at which an error occurred. Values in parentheses indicate the standard error of the mean (means calculated across sessions).

Element Sequence 1 Sequence 2

Avg. error trials % Avg. error trials %

I 11.31 ({plus minus} 0.82) 7.00 ({plus minus} 0.50) 22.15 ({plus minus} 1.50) 12.81 ({plus minus} 0.74)

II 2.90 ({plus minus} 0.40) 1.95 ({plus minus} 0.27) 1.77 ({plus minus} 0.29) 1.19 ({plus minus} 0.19)

III 0.44 ({plus minus} 0.13) 0.31 ({plus minus} 0.09) 0.64 ({plus minus} 0.15) 0.45 ({plus minus} 0.10)

IV 1.77 ({plus minus} 0.31) 1.19 ({plus minus} 0.20) 1.18 ({plus minus} 0.17) 0.83 ({plus minus} 0.12)

5. Line 213: since the stimuli within one sequence are interconnected, i.e. the response to the first element predicts responses to subsequent elements, shouldn’t we expect that the neural responses are also not independent within one trail. Then, strictly speaking, treating them as independent in the ANOVA would be not correct.

It is possible that the firing rates within a trial could be dependent. However, the spiking within the different intervals we are testing (i.e., firing rates for the different elements of a sequence) happens temporally far removed from each other (>700 ms difference between tested intervals), and does not follow a precise temporal offset (i.e., trial specific variation/jitter in the animal’s response to the target element introduced some additional random offset that was accounted for by alignment to element onset). Furthermore, the spiking appears as consistent across trials (see raster plots of Fig. 2). Therefore, we think it is fair to treat the firing rates during these disjoint intervals as independent.

Results:

If I understand it correctly, there are two possibilities when an error could occur at the element 1: if the sequence has been changed and the first element should be used by the bird as a cue so they are deemed to make an error; if the sequence has not been changed and the error does not indicate the sequence switch. You analyse only the first type of errors, it I get it correctly. Was there any difference at the neural/behavioural level between these two types of errors?

Due to the very low number of within block errors we decided against a separate analysis of those trials. On the behavioral level we observed that when pigeons made a within block error they usually returned to the correct sequence in the following trial. The avg. number of errors following a within block error was 0.43 ({plus minus}0.05, pigeon 1) and 0.52 ({plus minus}0.03, pigeon 2). In contrast, when errors occurred at block transitions (i.e., error feedback was necessary for stop and switch) pigeons had the tendency to repeat the erroneous response, before fully switching. In that case the avg. number of trials to switch from one sequence to the other was 2.77 {plus minus} 0.20 (pigeon 1) and 3.33 {plus minus} 0.20 (pigeon 2).

We differentiated between these types of error trials for our analysis and only analyzed the switch signal in the trials where the actual block switch occurred. Reasoning for this was that only the block switches triggered the desired executive behavioral switch that we wanted to investigate.

We have added the information about the within block errors to the manuscript (lines 280 ff.).

Figure 2: In the example of the NIML neuron from the figure 2 it is not clear, what element of the sequence this particular neuron is responding to (it might be element 3, or both 2 and 3). Did you perform post-hoc analysis to quantify how many of your seq-ele neurons actually code for one specific element?

We did not perform such post-hoc tests. As you correctly note it is unclear which element the example neuron is encoding with its differential firing rate. However, we think that for the scope of our study a specific encoding type (such as an element specific firing rate) is not essential. Our goal was to show that both regions, at the population level, do encode precise information of specific elements (i.e., the sequence-element interaction). We think we have shown this to be the case with our analysis of this information at the population level.

In contrast, describing the complex temporal encoding of elements at the single neuron level, shown in the two example neurons that have increased/decreased firing rates for multiple elements across both sequences at different time points, would require a clear hypothesis on what kind of encoding principle might be at play. Furthermore, we are not convinced that simple post-hoc testing would clarify this, as neurons appear to have a complex and potentially mixed selectivity.

Line 323: by “combinations of the factors” do you mean that their firing rate depends on at least one of the factors or an interaction of them?

We mean that those neurons are driven by any two combinations the factors (e.g., neuron is significant for factor element and for factor sequence), we have clarified this in the text (line 333 f.).

Figure 3: The amount of neurons that potentially encode the sequence is very low (<5% ?). Is it still above the false positive rate? Maybe you could create a table with ANOVA results and the number of corresponding neurons for each category? Or at least add the description of single bar graphs into the legend, because it is rather confusing now. For example, are neurons from “only element” group also included in the “only seq & elem” group as well?

We want to apologize for what was a confusing and insufficient labeling of the groups depicted in Fig. 3A, we have tried to clarify it, by splitting the figure up and showing the more detailed parts in a table (Review Tab. 2/Extended Data Figure 3-1). The labels of the bars meant to indicate that the respective factor became significant in an ANOVA. We tested the different factors that played a role in the task. The factors stimulus layout, target location and trial outcome affected firing rate of a substantial number of neurons. Independent of this, a 2-way ANOVA of the factors sequence and element (including their interaction) also affected firing rates of many neurons. We then looked into detailed combinations finding that few neurons were driven exclusively by the main factors (sequence, element) but many by the interaction (in combination with significance for at least one more main factor). Of those we found that some were driven by the interaction but not by the factors stimulus or sequence.

Review Table 2: Percentage of significant neurons by factor and different factor-combinations. Cells indicate the proportions of significant neurons. There was one two-way ANOVA (sequence and element, seq-ele interaction) and four one-way ANOVAs (visual stimulus, response location, outcome, respectively). The bottom most row denotes the percentage of neurons significant for the seq-ele interaction while having no significance for neither visual stimulus nor response location.

Significant factor NCL NIML

Neurons % Neurons %

Secondary factors

(each from a one way ANOVA) Stimulus 54 49.09 68 44.74

Location 68 61.82 109 71.71

Outcome 66 60.00 90 59.21

Main factors

(2-way ANOVA with factors sequence, element and their interaction) Only Sequence 4 3.64 8 5.26

Only Element 10 9.09 14 9.21

Only Interaction 11 10.00 10 6.58

Only seq & element 10 9.09 10 6.58

Interaction and any main 55 50.00 93 61.18

Of the above without significance for ‘stimulus’ or ‘location’ 19 17.27 24 15.79

Figure 4: After an error the white screen appeared for 2 seconds. How do you know that the neural response to the error is not the response to the visual stimulus (white screen)?

It might be a response to the white screen or a more abstract evaluative error. We have clarified in the discussion that we cannot disambiguate the two possibilities (lines 439 ff.).

The example neuron in NIML has a strong response at the end of the analysed period in the correct trials of the first sequence. Where does this response come from?

We cannot definitively determine the cause; it might be related to the ongoing block of trials, possibly a return to baseline.

Discussion:

Lines 480-482: While authors did such an effort to record simultaneously from two forebrain regions, I found it rather disappointing that the only analysis they provide is the latency of neural responses. To compare the latencies no simultaneous recording is needed. Instead, it would be great to see some cross-correlation analyses to maybe identify neurons that get direct input from NIML to NCL. I assume that authors actually did perform this analysis, but such direct neuron-to-neuron responses are obviously very challenging to find. So if this is the case, I would suggest that authors share this part of the analysis as well. It wouldn’t diminish the quality of their study, but actually help other people to appreciate the difficulty of the method. Therefore, I do not fully agree with the title which includes words “executive network”, because authors did neither provide a network analysis, nor did they analyse the full network.

Cross correlational analysis of spike trains of simultaneously recorded neurons did not yield any significant results. We appreciate the reviewer’s suggestion to include such analytical results. However, we remain hesitant to do so, as these negative findings cannot be interpreted in a meaningful way. Since they may depend on not-meaningful causes such as the rather low number of simultaneously recorded neurons, lack of direct targeting of connected neurons etc. Adding a full discussion of these points seems to unnecessarily bloat the manuscript. However, in order to highlight this open issue we have decided to add considerations to the discussion to point out that network analysis between the two regions is an important and worthwhile challenge (starting in line 524). We will pursue this further in the future.

Following your valuable comments on the issue, and in accordance with the comments of reviewer 2 we have also adjusted the title of the manuscript to focus more on the presence of two ‘executive regions’ in the pigeon brain, instead of claiming the presence of a ‘network’.

R2 --------------------------------------------------------------------------------------------------------------------------------------

Line 75: as a “stop-change protocol” may not be a very familiar procedure (it was not to me), possible expand with a few more words

Has been changed (lines 71 ff.)

Line 77: did you mean “event” or “extent”

Has been changed to ‘extent’.

Line 114: change “ventilating fan-generated” to “ventilate fan generated”

Has been changed.

Line 210: you tested the factor of response location. I was a bit unsure what “location” refers to. Do you mean left or right in the horizontal display, and up or down in the vertical display?

All four possible locations on screen are referred to as distinctive locations. We have further clarified this in figure caption 1 (line 291 f.) and in the methods section (lines 211 f.).

Line 314-315: You have a cell that fires to the second element of sequence 1 and that it can’t be explained by reward proximity, stimulus, or response location. The second element of sequence 1 is a response to the left on a horizontal display. The response to the left of the horizontal display also occurs in the fourth element of sequence 2. When I look at Figure 2 on the left it definitely fires to the fourth element of sequence 2, so to me this is not a cell that codes sequential information but rather a cell that simply fires to the left location on the horizontal display. It is very clear from the figure. How do you counter that argument?

We agree that there appears to be a small increase in firing rate for both red and blue, and we do acknowledge that location may also play a role in driving firing rates of some neurons. We gave an overview of the results of the ANOVA, testing the factor location that showed that a substantial number of neurons were affected by location (Review Tab. 2, formerly Fig. 3A (see also your comment below)).

However, the firing rates to element II in seq 1 (i.e., left location, color coded red) and the response to element IV in seq 2 (i.e., left location, color coded blue) are very different. We have juxtaposed this difference in firing rates for the left location in sequence 1 and 2 and quantified it (Review Fig. 2). The substantial difference between the sequences (and following from that the large effect size of the factor sequence) indicates that location by itself is not encoded in the firing rate, if it were then there should not be a difference between sequences. Instead, the combination (interaction) of sequence and location/element is what differentiates firing rate (also see response to the next comment).

Review Figure 2: Firing rates of the example neuron of NCL from Fig. 2, juxtaposition of left locations in sequences 1 and 2. There was a substantial difference in firing rates for the same location, depending on the sequence. The yellow line indicates the effect size of the factor sequence.

Similarly, the NIML cell in Figure 2 fires to the third element of sequence 2 which is the same position as the first element of sequence 2, and it certainly fires to the first element of sequence 2 as well. True, it does so in an inhibitory manner, but that is a response nonetheless. Again, to me this is a cell that fires to the bottom location of a vertical display. And as you noted in Figure 3a, most of your cells are indeed location cells.

We agree that in both cases the neuron responds to a location, but the nature of the response depends, not on the identity of the location, but on other variables (sequence/element). We believe that ignoring the direction of the neural response can lead to a misleading way to think about neural activity/coding. If the neural response to an identical stimulus differs significantly, in this case even in the sign (increase vs. decrease), it implies that this stimulus (location) is NOT a critical variable that explains neural activity. Instead, we argue that the fact that the neuron shows the opposite response to the same location, depending on sequence/element is a very strong case for a neural representation of element.

This is what we have quantified as the interaction and what we consider the most relevant finding (please refer to our response to the next comment for more details).

Figure 3a: To me, the number of cells that yielded a significant factor of sequence, or sequence and element, are around 5%. Given the large number of tests you conducted, would you not expect about 5% to come out significant that weren’t?

We want to apologize for the layout of Fig. 3a, it seems to have caused more confusion than it helped. Our intention was to give an overview of all relevant factors that played a role in the task, and how neuronal activity was affected by them.

We consider the subset of neurons that were significant for the interaction between sequence and element without significance for the confounding factors stimulus to be the most important (i.e., ‘interaction not stim or loc’). These kinds of neurons were observed at equal proportions in NCL and NIML. We argue that firing rates of these neurons encode the precise identity of the current element.

There was a large proportion of neurons that showed significance for the interaction of sequence with element that was also significantly affected by the factors stimulus and location (i.e., those labelled ‘int & main’). We show the proportions of those neurons separately (i.e., ‘stimulus’, ‘location’).

The bars labelled with ‘only sequence’ etc. showed the small proportions of neurons that were exclusively affected by those factors (i.e., no interaction).

To highlight the complex neuronal activity of the ‘interaction neurons’ we had quantified the number of neurons that was driven by the interaction but not by the factors location or stimulus. We found that even when we accounted for those factors there was a substantial number of significant neurons

We have removed the bar plot that was evidently causing confusion from the figure and added a table for a better overview of the statistical results (Review Table 2/Extended Data Figure 3-1).

Review Table 2: Percentage of significant neurons by factor and different factor-combinations. Cells indicate the proportions of significant neurons. There was one two-way ANOVA (sequence and element, seq-ele interaction) and four one-way ANOVAs (visual stimulus, response location, outcome, respectively). The bottom most row denotes the percentage of neurons significant for the seq-ele interaction while having no significance for neither visual stimulus nor response location.

Significant factor NCL NIML

Neurons % Neurons %

Secondary factors

(each from a one way ANOVA) Stimulus 54 49.09 68 44.74

Location 68 61.82 109 71.71

Outcome 66 60.00 90 59.21

Main factors

(2-way ANOVA with factors sequence, element and their interaction) Only Sequence 4 3.64 8 5.26

Only Element 10 9.09 14 9.21

Only Interaction 11 10.00 10 6.58

Only seq & element 10 9.09 10 6.58

Interaction and any main 55 50.00 93 61.18

Of the above without significance for ‘stimulus’ or ‘location’ 19 17.27 24 15.79

Figure 3b: On my computer, the element legend is blue, but the element graph is purple.

We have changed the color labels to fit.

To me, your data are clear that there are no difference between NCL and NIML in terms of the response profiles. The only difference is a slightly earlier, in the region of around 200 msec, emergence of a neural response in NIML, and the fact that the PEV for outcome was higher in NCL than NIML. Two comments.

1. One comment is whether what we are dealing with raises the issue of statistically significant vs statistically meaningful. In brain terms 200 msec is quite long, I agree, but I wonder whether connections/anatomy can explain the difference, rather than differences in some form of sequential processing.

2. Second, a complex story is created around this difference in terms of what the NCL and NIML might be contributing to sequential behaviour. Given the similarities between the areas on so many other dimensions, is there no other, simpler, interpretation of the contribution of these regions to sequential behaviour other than outcome monitoring? To me the fact that you have identified another potential executive area in the avian brain is good enough.

Thank you for these comments. To our knowledge the two regions are connected directly, without further relaying regions. However, knowledge on this subject is sparse. We think (and have further emphasized this in the manuscript) that it is important that NIML and connected regions are given further attention with additional studies that focus in detail on specific aspects of executive control (like the switching behavior).

We have edited the manuscript to shift the focus on the similarities between regions and to stress that NIML could be an additional candidate for an executive region.

Following your critique, in combination with the comments of reviewer 1, we have also adjusted the title of the manuscript to focus more on the presence of two ‘executive’ regions in the pigeon brain, instead of claiming the presence of a ‘network’.
