# Supplementary material for: Executive Control of Sequence Behavior in Pigeons Involves Two Distinct Brain Regions
Source: eNeuro. 2023 Mar 3;10(3):ENEURO.0296-22.2023. doi: 10.1523/ENEURO.0296-22.2023 (PMC9997693; doi:10.1523/ENEURO.0296-22.2023)
Supplement: Extended Data Figure 3-1 — Percentage of significant neurons by factor and different factor combinations. Cells indicate the proportions of significant neurons. There was one two-way ANOVA (sequence, and element, seq–ele interaction) and four one-way ANOVAs (visual stimulus, response location, outcome, respectively). The bottom most row denotes the percentage of neurons significant for the seq–ele interaction while having no significance for either visual stimulus or response location. Download Figure 3-1, DOC file. [file enu-eN-NWR-0296-22-s03.doc]

|  | **Significant factor** | **NCL** | | **NIML** | |
| --- | --- | --- | --- | --- | --- |
|  |  | **Neurons** | **%** | **Neurons** | **%** |
| **Secondary factors**  **(each from a one way ANOVA)** | **Stimulus** | 54 | 49.09 | 68 | 44.74 |
|  | **Location** | 68 | 61.82 | 109 | 71.71 |
|  | **Outcome** | 66 | 60.00 | 90 | 59.21 |
| **Main factors**  **(2-way ANOVA with factors sequence, element and their interaction)** | **Only Sequence** | 4 | 3.64 | 8 | 5.26 |
|  | **Only Element** | 10 | 9.09 | 14 | 9.21 |
|  | **Only Interaction** | 11 | 10.00 | 10 | 6.58 |
|  | **Only seq & element** | 10 | 9.09 | 10 | 6.58 |
|  | **Interaction and any main** | 55 | 50.00 | 93 | 61.18 |
|  | **Of the above without significance for ‘stimulus’ or ‘location’** | 19 | 17.27 | 24 | 15.79 |
