# Supplementary material for: Executive Control of Sequence Behavior in Pigeons Involves Two Distinct Brain Regions
Source: eNeuro. 2023 Mar 3;10(3):ENEURO.0296-22.2023. doi: 10.1523/ENEURO.0296-22.2023 (PMC9997693; doi:10.1523/ENEURO.0296-22.2023)
Supplement: Extended Data Figure 4-1 — Overview of error trials. Average number of errors and percentage of errors of all trials for sequences 1 and 2, sorted by the element at which an error occurred. Values in parentheses indicate the SEM (means calculated across sessions). Download Figure 4-1, DOC file. [file enu-eN-NWR-0296-22-s04.doc]

| Element | Sequence 1 | | Sequence 2 | |
| --- | --- | --- | --- | --- |
|  | Avg. error trials | *%* | Avg. error trials | *%* |
| I | 11.31 (± 0.82) | *7.00 (± 0.50)* | 22.15 (± 1.50) | *12.81 (± 0.74)* |
| II | 2.90 (± 0.40) | *1.95 (± 0.27)* | 1.77 (± 0.29) | *1.19 (± 0.19)* |
| III | 0.44 (± 0.13) | *0.31 (± 0.09)* | 0.64 (± 0.15) | *0.45 (± 0.10)* |
| IV | 1.77 (± 0.31) | *1.19 (± 0.20)* | 1.18 (± 0.17) | *0.83 (± 0.12)* |
