# Supplementary material for: Executive Control of Sequence Behavior in Pigeons Involves Two Distinct Brain Regions
Source: eNeuro. 2023 Mar 3;10(3):ENEURO.0296-22.2023. doi: 10.1523/ENEURO.0296-22.2023 (PMC9997693; doi:10.1523/ENEURO.0296-22.2023)
Supplement: Extended Data Figure 4-2 — Statistical results of t test for outcome PEV. Download Figure 4-2, DOC file. [file enu-eN-NWR-0296-22-s05.doc]

| **Bin time rel. to response** | ***t*** | ***df*** | ***p*** | ***d*** |
| --- | --- | --- | --- | --- |
| -1000 | -0.9364 | 260 | 0.3499 | 0.1223 |
| -900 | -0.9527 | 260 | 0.3416 | 0.125 |
| -800 | -1.0403 | 260 | 0.2992 | 0.1346 |
| -700 | -1.1653 | 260 | 0.245 | 0.1492 |
| -600 | -1.6428 | 260 | 0.1016 | 0.2108 |
| -500 | -1.6761 | 260 | 0.0949 | 0.2148 |
| -400 | -1.9543 | 260 | 0.0517 | 0.2523 |
| -300 | -1.5289 | 260 | 0.1275 | 0.1979 |
| -200 | -1.0551 | 260 | 0.2924 | 0.137 |
| -100 | -2.6569 | 260 | 0.0084 | 0.3472 |
| 0 | -1.8047 | 260 | 0.0723 | 0.2283 |
| 100 | -0.9781 | 260 | 0.3289 | 0.1216 |
| 200 | -0.8412 | 260 | 0.401 | 0.1045 |
| 300 | -0.5728 | 260 | 0.5672 | 0.0711 |
| 400 | 0.8123 | 260 | 0.4174 | 0.0994 |
| 500 | 0.8318 | 260 | 0.4063 | 0.1029 |
| 600 | 0.6875 | 260 | 0.4924 | 0.0855 |
| 700 | 0.886 | 260 | 0.3764 | 0.1095 |
| 800 | 0.7573 | 260 | 0.4496 | 0.0928 |
| 900 | 0.4977 | 260 | 0.6191 | 0.0611 |
| 1000 | 0.7539 | 260 | 0.4516 | 0.0924 |
| 1100 | 0.9822 | 260 | 0.3269 | 0.1191 |
| 1200 | 0.9994 | 260 | 0.3185 | 0.1199 |
| 1300 | 1.1062 | 260 | 0.2696 | 0.1321 |
| 1400 | 1.4473 | 260 | 0.149 | 0.1723 |
| 1500 | 2.0745 | 260 | 0.039 | 0.2447 |
| 1600 | 2.2998 | 260 | 0.0223 | 0.2698 |
| 1700 | 2.8834 | 260 | 0.0043 | 0.3375 |
| 1800 | 3.4444 | 260 | 0.0007 | 0.402 |
| 1900 | 3.6923 | 260 | 0.0003 | 0.43 |
| 2000 | 3.8713 | 260 | 0.0001 | 0.4503 |
| 2100 | 4.0015 | 260 | 0.0001 | 0.4647 |
| 2200 | 3.6753 | 260 | 0.0003 | 0.426 |
| 2300 | 3.4508 | 260 | 0.0007 | 0.3998 |
| 2400 | 3.2101 | 260 | 0.0015 | 0.3732 |
| 2500 | 2.845 | 260 | 0.0048 | 0.3326 |
| 2600 | 2.3738 | 260 | 0.0183 | 0.2802 |
| 2700 | 2.075 | 260 | 0.039 | 0.2462 |
| 2800 | 2.3319 | 260 | 0.0205 | 0.2755 |
| 2900 | 2.0859 | 260 | 0.038 | 0.2444 |
| 3000 | 1.7305 | 260 | 0.0847 | 0.2029 |
| 3100 | 1.7466 | 260 | 0.0819 | 0.2053 |
| 3200 | 1.4002 | 260 | 0.1627 | 0.1662 |
| 3300 | 1.4382 | 260 | 0.1516 | 0.1717 |
| 3400 | 1.1036 | 260 | 0.2708 | 0.1333 |
| 3500 | 1.302 | 260 | 0.1941 | 0.1573 |
| 3600 | 1.4387 | 260 | 0.1514 | 0.1726 |
| 3700 | 1.08 | 260 | 0.2811 | 0.1303 |
| 3800 | 0.5208 | 260 | 0.603 | 0.0632 |
| 3900 | -0.1454 | 260 | 0.8845 | 0.0179 |
| 4000 | -0.9055 | 260 | 0.366 | 0.114 |
| 4100 | -0.8397 | 260 | 0.4018 | 0.1053 |
| 4200 | -0.476 | 260 | 0.6345 | 0.06 |
| 4300 | -0.5754 | 260 | 0.5655 | 0.0729 |
| 4400 | -0.1301 | 260 | 0.8966 | 0.0162 |
| 4500 | 0.4097 | 260 | 0.6823 | 0.05 |
| 4600 | 0.5406 | 260 | 0.5893 | 0.0665 |
| 4700 | 0.6131 | 260 | 0.5403 | 0.0753 |
| 4800 | 0.7276 | 260 | 0.4675 | 0.0899 |
| 4900 | 0.1456 | 260 | 0.8843 | 0.0183 |
| 5000 | -0.9364 | 260 | 0.3499 | 0.1223 |
