# Supplementary material for: Executive Control of Sequence Behavior in Pigeons Involves Two Distinct Brain Regions
Source: eNeuro. 2023 Mar 3;10(3):ENEURO.0296-22.2023. doi: 10.1523/ENEURO.0296-22.2023 (PMC9997693; doi:10.1523/ENEURO.0296-22.2023)
Supplement: Extended Data Figure 4-4 — Significant time bins (in the interval –2000 to 1000 ms relative to sequence initiation) based on results of the permutation for switch (1000 permutations; significant if probability was <2.5%) for the whole population. Bin width, 100 ms. Download Figure 4-4, DOC file. [file enu-eN-NWR-0296-22-s07.doc]

| **Region** | **Bin start time** | **Significant factor** | **Probability of permutated result larger (%)** |
| --- | --- | --- | --- |
| NIML | -100 | switch | < 0.1 |
| NIML | 0 | switch | 0.3 |
