# Supplementary material for: Executive Control of Sequence Behavior in Pigeons Involves Two Distinct Brain Regions
Source: eNeuro. 2023 Mar 3;10(3):ENEURO.0296-22.2023. doi: 10.1523/ENEURO.0296-22.2023 (PMC9997693; doi:10.1523/ENEURO.0296-22.2023)
Supplement: Extended Data Figure 4-5 — Significant time bins (in the interval –2000 to 1000 ms relative to sequence initiation) based on the results of the permutation for switch (1000 permutations; significant if the probability was <2.5%) for the subpopulation. Bin width, 100 ms. Download Figure 4-5, DOC file. [file enu-eN-NWR-0296-22-s08.doc]

| **Region** | **Bin start time** | **Significant factor** | **Probability of permutated result larger (%)** |
| --- | --- | --- | --- |
| NCL | -200 | switch | 0.2 |
| NCL | -100 | switch | 0.2 |
| NCL | 300 | switch | 1.1 |
| NCL | 400 | switch | 0.8 |
| NIML | -300 | switch | 0.2 |
| NIML | -200 | switch | 0.1 |
| NIML | -100 | switch | < 0.1 |
| NIML | 0 | switch | < 0.1 |
| NIML | 800 | switch | 2.2 |
| NIML | 900 | switch | 0.5 |
| NIML | 400 | interaction | 0.3 |
| NIML | 500 | interaction | < 0.1 |
