# Supplementary material for: Executive Control of Sequence Behavior in Pigeons Involves Two Distinct Brain Regions
Source: eNeuro. 2023 Mar 3;10(3):ENEURO.0296-22.2023. doi: 10.1523/ENEURO.0296-22.2023 (PMC9997693; doi:10.1523/ENEURO.0296-22.2023)
Supplement: Extended Data Figure 4-6 — Statistical results of t test for factor switch PEV between NCL and NIML subpopulations. Download Figure 4-6, DOC file. [file enu-eN-NWR-0296-22-s09.doc]

| **Bin time rel. to response** | ***t*** | ***df*** | ***p*** |
| --- | --- | --- | --- |
| -3000 | 1.4341 | 37 | 0.1599 |
| -2900 | -2.224 | 34 | 0.0329 |
| -2800 | 0.2771 | 37 | 0.7833 |
| -2700 | 1.5012 | 38 | 0.1416 |
| -2600 | -0.0795 | 38 | 0.9371 |
| -2500 | -2.1408 | 36 | 0.0391 |
| -2400 | 0.4137 | 38 | 0.6814 |
| -2300 | -1.6724 | 37 | 0.1029 |
| -2200 | -0.466 | 36 | 0.6441 |
| -2100 | -0.9096 | 37 | 0.3689 |
| -2000 | -1.5057 | 38 | 0.1404 |
| -1900 | -1.6948 | 37 | 0.0985 |
| -1800 | -0.7847 | 37 | 0.4376 |
| -1700 | -0.6329 | 37 | 0.5307 |
| -1600 | -0.1316 | 38 | 0.896 |
| -1500 | -0.9974 | 39 | 0.3247 |
| -1400 | -0.5357 | 39 | 0.5952 |
| -1300 | -0.8262 | 39 | 0.4137 |
| -1200 | -2.1015 | 39 | 0.0421 |
| -1100 | -1.4758 | 38 | 0.1482 |
| -1000 | -0.2202 | 36 | 0.8269 |
| -900 | -0.3315 | 37 | 0.7421 |
| -800 | -1.096 | 37 | 0.2801 |
| -700 | -1.4961 | 37 | 0.1431 |
| -600 | -0.7475 | 35 | 0.4598 |
| -500 | -0.5026 | 38 | 0.6181 |
| -400 | 1.1767 | 39 | 0.2464 |
| -300 | -1.3749 | 37 | 0.1774 |
| -200 | 0.0342 | 36 | 0.9729 |
| -100 | -0.2046 | 35 | 0.8391 |
| 0 | -1.4659 | 35 | 0.1516 |
| 100 | -1.8489 | 36 | 0.0727 |
| 200 | -0.2252 | 34 | 0.8231 |
| 300 | 0.0902 | 36 | 0.9286 |
| 400 | 0.5548 | 34 | 0.5826 |
| 500 | -0.433 | 33 | 0.6678 |
| 600 | -0.8201 | 36 | 0.4176 |
| 700 | -1.2592 | 37 | 0.2158 |
| 800 | -1.4059 | 34 | 0.1688 |
| 900 | -0.9516 | 35 | 0.3478 |
